# Palladium-catalyzed ring-opening reactions of cyclopropanated 7-oxabenzonorbornadiene with alcohols

**DOI:** 10.3762/bjoc.12.209

**Published:** 2016-10-14

**Authors:** Katrina Tait, Oday Alrifai, Rebecca Boutin, Jamie Haner, William Tam

**Affiliations:** 1 Guelph-Waterloo Centre for Graduate Work in Chemistry and Biochemistry, Department of Chemistry, University of Guelph, Guelph, Ontario, N1G 2W1, Canada

**Keywords:** alcohol nucleophiles, C1 substitution, cyclopropanated oxabenzonorbornadiene, palladium catalysis, ring-opening reactions

## Abstract

Palladium-catalyzed ring-opening reactions of cyclopropanated 7-oxabenzonorbornadiene derivatives using alcohol nucleophiles were investigated. The optimal conditions were found to be 10 mol % PdCl_2_(CH_3_CN)_2_ in methanol, offering yields up to 92%. The reaction was successful using primary, secondary and tertiary alcohol nucleophiles and was compatible with a variety of substituents on cyclopropanated oxabenzonorbornadiene. With unsymmetrical C1-substituted cyclopropanated 7-oxabenzonorbornadienes, the regioselectivity of the reaction was excellent, forming only one regioisomer in all cases.

## Introduction

**Scheme 1:**
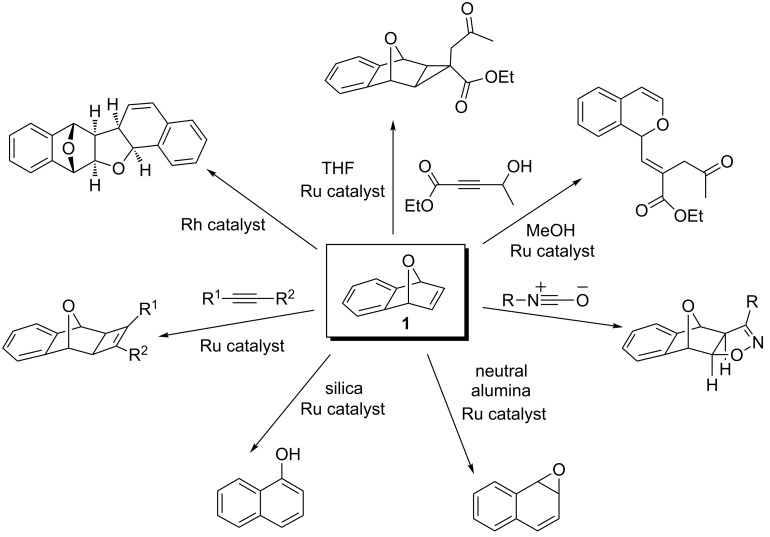
Various chemical transformations of 7-oxabenzonorbornadiene **1**.

**Scheme 2:**
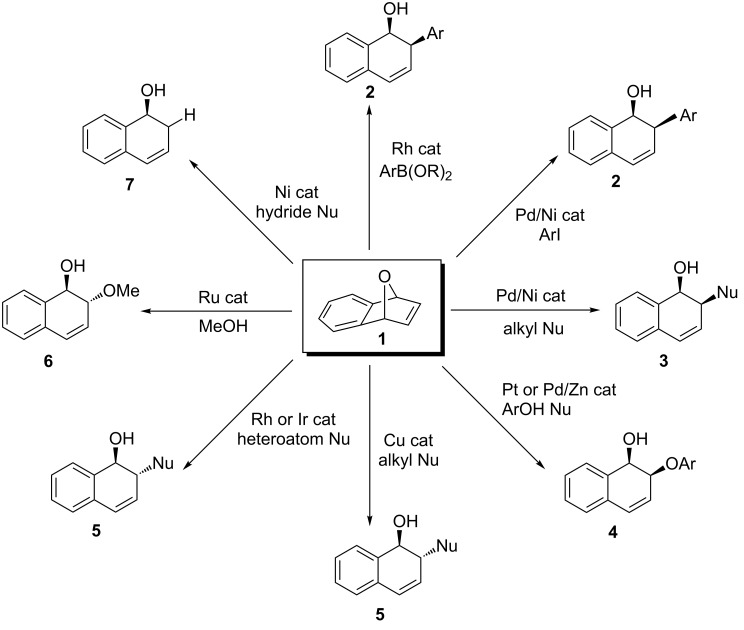
Nucleophilic ring-opening reactions of 7-oxabenzonorbornadiene **1**.

Heterobicyclic alkenes undergo important chemical transformations to provide highly substituted cyclic and acyclic systems [1–2]. Oxabicyclic alkene **1** specifically can undergo a variety of chemical transformations to generate highly substituted and complex organic frameworks (Scheme 1) [3–13]. An important chemical transformation is the nucleophilic ring opening of oxabicyclic alkene **1**, which offers a diverse collection of dihydronaphthalenols depending on the metal catalyst and nucleophiles used (Scheme 2). *Syn-*stereoisomeric products **2** and **3** can be obtained using rhodium [14], palladium [15], or nickel [16] catalysts with an arene nucleophile and when palladium [17] or nickel [18] are used with an alkyl nucleophile. Recently, it was shown that the *syn*-stereoisomeric product **4** could be obtained through the use of platinum catalysts [19] or palladium catalysts with zinc co-catalyst with phenol nucleophiles [20]. Meanwhile, *anti-*stereoisomeric products **5** and **6** are obtained when copper catalysts are used with alkyl nucleophiles [21], if rhodium [22] or iridium catalysts are used in the presence of heteroatomic nucleophiles [23–24], or when ruthenium catalysts are used with alcohol nucleophiles [25]. Furthermore, unsubstituted dihydronaphthalenols **7** can be obtained through the reductive ring opening of oxabicyclic alkene **1** with hydride nucleophiles [26]. These intermediates find synthetic uses in the preparation of biologically active substances such as arnottin I [27] and sertraline [28].

**Scheme 3:**
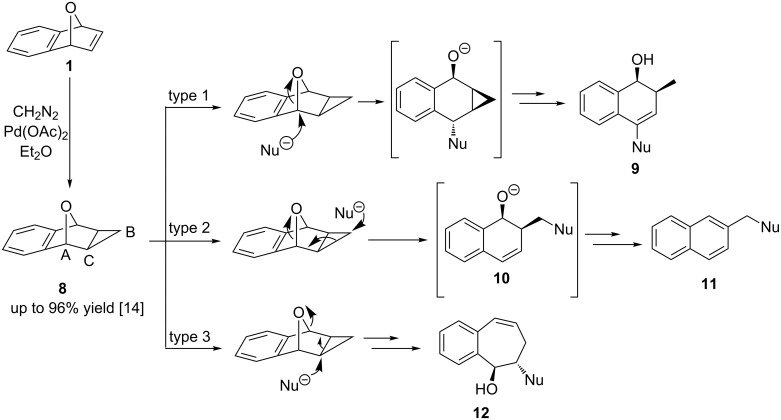
Preparation of cyclopropanated **8** and its proposed ring-opening mechanisms.

While the nucleophilic ring openings of oxabenzonorbornadiene **1** have been extensively studied, no examples of a metal-catalyzed ring opening of cyclopropanated compound **8** have been reported in the literature. Oxabenzonorbornadiene **1** and its derivatives are first cyclopropanated with diazomethane under palladium catalysis to afford **8** in good to excellent yields [29]. Cyclopropanated **8** has been predicted to undergo three distinct ring-opening mechanisms (Scheme 3). The first ring-opening type (type 1) involves the attack of the nucleophile at bridgehead carbon A, resulting in cleavage of the C–O bond. Through deprotonation at the bridgehead position and an internal rearrangement, 2-methyldihydronaphthalen-1-ols **9** could be formed. This type 1 ring opening has been accomplished by our group through the use of organocuprate nucleophiles [30]. The second type of predicted ring opening (type 2) involves the attack of the nucleophile at the external cyclopropane carbon B, resulting in the cleavage of the cyclopropane C–C bond followed by a C–O bond cleavage to produce 2-substituted dihydronaphthalenols **10**. Under thermal conditions, the dihydronaphthalenols can fully aromatize to form various substituted naphthalene derivatives **11**. This has been accomplished through acid catalysis with various alcohol nucleophiles [31]. The last type of predicted ring opening (type 3) which has not yet been observed involves the attack of the nucleophile at the internal cyclopropane carbon C, which could induce ring expansion to form seven-membered ring **12** .

In this paper, we aim to explore the use of a palladium catalyst with an alcohol nucleophile on the ring opening of cyclopropanated oxabenzonorbornadiene with the goal of determining which type of ring-opening pathway it follows. This complements previous studies by our group involving the ring opening of cyclopropanated oxabenzonorbornadiene through the novel use of a transition metal catalyst. Using a transition metal catalyst could reveal new ring-opening pathways and provide further insight into the reactivity of strained cyclopropanated oxabicyclic compounds.

## Results and Discussion

The effect of different palladium catalysts and catalyst equivalency were first investigated, with the results summarized in Table 1. In the presence of a palladium(0) catalyst (Table 1, entries 1–3), the reaction did not proceed and the starting material was recovered. The effect of a palladium(II) catalyst was then investigated (Table 1, entries 4–9), producing variable yields of substituted naphthalene **11a**. While attempts using Pd(OAc)_2_ (Table 1, entry 4) and PdCl_2_(PPh_3_)_2_ (Table 1, entry 5) were unsuccessful, palladium(II) catalysts in the absence of a triphenylphosphine ligand were more promising (Table 1, entries 6–9). The palladium(II) catalyst PdCl_2_(CH_3_CN)_2_ generated a high yield of substituted naphthalene **11a** after only 24 hours and was chosen to further optimize reaction conditions. When the catalyst equivalency was investigated, lowering the catalyst from 10 mol % to 5 mol % reduced the yield from 89% to 27% (Table 1, entry 10) while further reducing the catalyst equivalency to 2 mol % showed no reaction (Table 1, entry 11). To confirm that the presence of a triphenylphosphine ligand would result in no reaction, the optimized catalyst was used with an external source of triphenylphosphine, which resulted in no reaction (Table 1, entry 12). To expand the scope of catalyst, the effect of using a platinum catalyst was investigated. The use of a platinum(IV) catalyst resulted in no reaction (Table 1, entry 13) along with the use of a platinum(II) catalyst (Table 1, entry 14). Using an anionic platinum(II) catalyst yielded substituted naphthalene **11a** in a 22% yield, though this was considerably lower when compared to the optimized palladium catalyst.

**Table 1:** Effects of palladium catalysts and catalyst equivalency on the ring-opening reaction of oxabicyclic alkene **8a** with alcohols.

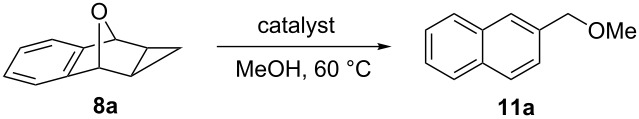				

Entry^a^	Catalyst	Catalyst (mol %)	Time (h)	Yield (%)^b^

1	Pd(PPh_3_)_4_	10	144	0^c^
2	Pd_2_(dba)_3_	10	144	0^c^
3	Pd/C	10	144	0^c^
4	Pd(OAc)_2_	10	72	0^c^
5	PdCl_2_(PPh_3_)_2_	10	72	0^c^
6	PdCl_2_(dppf)	10	48	87
7	PdCl_2_	10	24	89
8	PdCl_2_(PhCN)_2_	10	48	90
9	PdCl_2_(CH_3_CN)_2_	10	24	89
10	PdCl_2_(CH_3_CN)_2_	5	72	27
11	PdCl_2_(CH_3_CN)_2_	2	48	0^c^
12	PdCl_2_(CH_3_CN)_2_ + PPh_3_	10	216	0^c^
13	PtO_2_	10	72	0^c^
14	PtCl_2_	10	72	0^c^
15	K[(PtCl_3_CCH_2_=CH_2_)]·xH_2_O	10	48	22

^a^Reaction was completed on a 30 mg scale of **8a**. ^b^Isolated yield after column chromatography.^c^70–97% of **8a** was recovered.

A variety of solvents were next screened including polar aprotic, polar protic, and aromatic solvents (Table 2). The polar aprotic solvents DMSO, DMF, and acetonitrile (Table 2, entries 1–3) caused little or no reaction to occur. Polar aprotic solvents DCE and THF (Table 2, entries 4 and 7) saw good yield of naphthalene **11a**. The effect of nucleophile equivalency was investigated using THF, and when the equivalency was reduced to 10 equivalents (Table 2, entry 6), the yield decreased slightly to 78% while further decreasing the nucleophile equivalency to 5 equivalents (Table 2, entry 5) saw a very small further decrease to a 77% yield. The aromatic solvent toluene was investigated which saw a high yield of 92% (Table 2, entry 10) so the effect of nucleophile equivalency was investigated. When the equivalency was decreased to 10 equivalents (Table 2, entry 9) the yield decreased to 85% while further decreasing the nucleophile equivalency to 5 equivalents decreased the yield to 71% (Table 2, entry 8). The polar protic solvent methanol was investigated since it is also a nucleophile and showed a high yield of 89% (Table 2, entry 11). Using methanol, the effect of temperature was investigated. Decreasing the temperature to 40 °C resulted in a reduction of yield to 70% (Table 2, entry 12) while further lowering the temperature resulted in no reaction (Table 2, entry 13).

**Table 2:** Effects of solvent, nucleophile equivalency, and temperature on the ring-opening reactions of **8a** with alcohols.

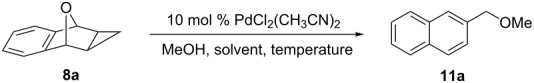					

Entry^a^	Solvent	MeOH equivalency	Temperature (°C)	Time (h)	Yield (%)^b^

1	DMSO	20	60	24	0^c^
2	DMF	20	60	120	0^c^
3	CH_3_CN	20	60	24	trace
4	DCE	20	60	24	82
5	THF	5	60	24	77
6	THF	10	60	24	78
7	THF	20	60	24	82
8	toluene	5	60	24	71
9	toluene	10	60	24	85
10	toluene	20	60	24	92
11	MeOH	20	60	24	89
12	MeOH	20	40	168	70
13	MeOH	20	25	48	0^c^

^a^Reaction was completed on a 30 mg scale of **8a**. ^b^Isolated yield after column chromatography. ^c^74–85% of **8a** was recovered.

The scope of the reaction was expanded to include type 2 ring openings of symmetrical substituted cyclopropanated 7-oxabenzonorbornadiene (Table 3). The effect of substituents at both bridgehead positions was first investigated. With a methyl group at both bridge head positions, the yield was decreased to 40% at 90 °C (Table 3, entry 1). Substitution on the arene portion of cyclopropanated oxabenzonorbornadiene **8a** was investigated. *p*-Methoxy-substituted **8c** underwent minimal conversion to the ring-opened product with a yield of only 5% (Table 3, entry 2). While no starting material was recovered, a complex mixture of products were observed. *o*-Methoxy-substituted **8d** was able to undergo ring opening to produce **11d** in a moderate yield of 46% (Table 3, entry 3). The effect of a halide substitution on the arene was also investigated in the *ortho* position which decreased the yield to 37% (Table 3, entry 4).

**Table 3:** Scope of the reaction with symmetrical substituted cyclopropanated 7-oxabenzonorbornadiene.

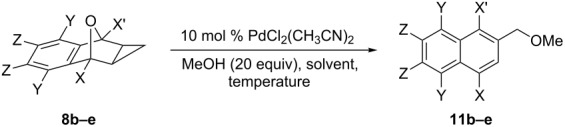								

Entry^a^	X	X’	Y	Z	Time (days)	Temperature (°C)	Solvent	Yield (%)^b^

1	Me	Me	H	H	10	90	toluene	40
2	H	H	OMe	H	14	90	toluene	5
3	H	H	H	OMe	14	90	toluene	46
4	H	H	H	Br	14	90	toluene	37

^a^Reaction was completed on a 30 mg scale (0.1–0.3 mmol) of **8b–e**. ^b^Isolated yield by column chromatography.

**Scheme 4:**
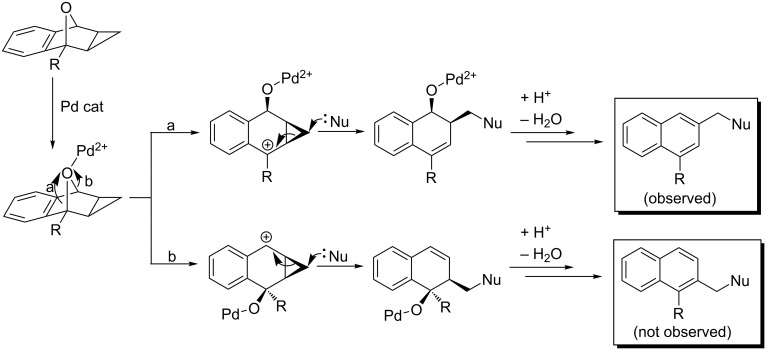
Formation of the possible regioisomers for the ring opening of asymmetric C1-substituted cyclopropanated oxabenzonorbornadiene.

The scope of the reaction was then extended to include examples of unsymmetrical functionalized substrates **8f–j** bearing substituents at the C1 position. With a substituent at the C1 position, the formation of two regioisomers is possible (Scheme 4). The bridgehead-oxygen bond can break in two different directions (a or b), creating either a tertiary or secondary cation which after the nucleophilic ring opening creates two different regioisomers. In all cases, the regioselectivity of this reaction is excellent, forming only one regioisomer. Compared with the reaction of unsubstituted **8a**, substitution at the C1 position significantly decreased the yield (Table 4). When the size of the substituent increases, the general trend is that the yield of the reaction decreases. Unexpectedly, with a methyl group at the C1 position, however, the yield was lower than with larger substituents at the C1 position with starting material still being recovered after one week. The reaction was repeated multiple times both at 60 °C (Table 4, entry 1) and 90 °C (Table 4, entry 2) and showed yields of only 27% and 41%, respectively. With an ethyl substituent at the C1 position, the yield decreased to 58% at 60 °C (Table 4, entry 3) or was marginally enhanced in toluene at 90 °C with a 65% yield though the reaction took almost twice as long (Table 4, entry 4). Increasing the steric bulk at the C1 position to a *tert*-butyl group decreased the yield further to 47% (Table 4, entry 5). Electron-withdrawing groups were then investigated at the C1 position and led to an appreciable reduction in conversion of **8a** to the corresponding ring-opened product. An acyl group at the C1 position caused the yield to decrease to 29% (Table 4, entry 6) while a methyl ester substituent at the C1 position further decreased the yield to 23% (Table 4, entry 7).

**Table 4:** Scope of the reaction with unsymmetrical substituted cyclopropanated 7-oxabenzonorbornadiene.

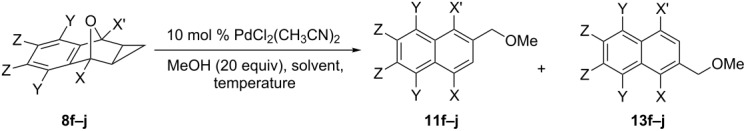									

Entry^a^	X	X’	Y	Z	Time (days)	Temperature (°C)	Solvent	Yield (%)^b^	Regioselectivity **11**:**13**

1	Me	H	H	H	7	60	MeOH	27^c^	100:0
2	Me	H	H	H	8	90	toluene	41^c^	100:0
3	Et	H	H	H	7	60	MeOH	58	100:0
4	Et	H	H	H	13	90	toluene	65	100:0
5	*t-*Bu	H	H	H	8	90	toluene	47	100:0
6	C(O)Me	H	H	H	7	90	toluene	29^c^	100:0
7	COOMe	H	H	H	14	90	toluene	23	100:0

^a^Reaction was completed on a 30 mg (0.1–0.3 mmol) scale of **8f–j**. ^b^Isolated yield by column chromatography. ^c^41–58% starting material recovered.

The scope of this reaction was also expanded to include different alcohol nucleophiles (Table 5). By using a primary alcohol nucleophile, a decrease in reactivity was seen with increasing chain length (Me < Et < *n-*Bu; Table 5, entries 1, 2 and 3) while maintaining reasonable yields in a short period of time. When 2-methoxyethanol was used, a good yield of 80% was observed, although the reaction took much longer to complete (Table 5, entry 4). Similarly, when isobutanol was investigated, the conversion to ring-opened product **11n** took 10 days but was still able to achieve a moderate yield of 60% (Table 5, entry 5). Using a secondary alcohol as the nucleophile resulted in an incomplete conversion to ring-opened product **11o** even after 25 days, with **8a** still recovered as an inseparable mixture (Table 5, entry 6). Unexpectedly, using a tertiary alcohol proceeded quicker than a secondary alcohol and resulted in complete conversion to ring-opened product **11p** in a moderate yield of 56% (Table 5, entry 7). Cyclic alcohol nucleophiles were also investigated, starting with cyclohexanol, which resulted in a moderate yield of 63% after only 1 day (Table 5, entry 8). When benzyl alcohol was used, no reaction occurred (Table 5, entry 9) and similarly when phenol was used, no reaction occurred and **8a** was recovered (Table 5, entry 10).

**Table 5:** Scope of reaction with different alcohol nucleophiles.

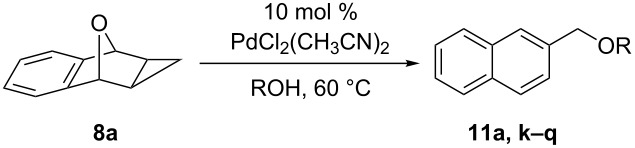			

Entry^a^	ROH^b^	Time (h)	Yield (%)^c^

1	MeOH	144	89
2	EtOH	144	85
3	*n*-BuOH	144	68
4	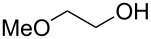	72	80
5	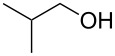	240	60
6	iPrOH	600	41^d,e^
7	*t*-BuOH	24	56
8	CyOH	48	63
9	BnOH	24	0^f^
10	PhOH	24	0^d,f^

^a^Reaction was completed on a 30 mg scale of **8a**. ^b^Alcohol was used as nucleophile and solvent. ^c^Isolated yield by column chromatography. ^d^6–58% of **8a** was recovered. ^e^Yield by ^1^H NMR. ^f^Reaction was attempted using toluene as a solvent at 90 °C but no reaction occurred.

## Conclusion

In conclusion, we have demonstrated the first examples of palladium-catalyzed type 2 ring-opening reactions of cyclopropanated oxabenzonorbornadienes with alcohols. The optimized conditions include PdCl_2_(CH_3_CN)_2_ with the alcohol nucleophile as the solvent at 60 °C or with toluene added at 90 °C to produce 2-substituted dihydronaphthalenols. The scope of the reaction was successfully expanded to include the ring opening of various symmetrical substituted cyclopropanated oxabenzonorbornadienes. When unsymmetrical substrates were investigated, the regioselectivity of the reaction was excellent, forming only one regioisomer in all cases. The scope of the reaction was also successfully expanded to include various primary, secondary, and tertiary alcohol nucleophiles.

## Supporting Information

Experimental procedures and copies of ^1^H and ^13^C NMR spectra for compounds **11d, g–i, m, n**.

File 1 Experimental.

File 2 NMR Spectra.
