# Supplementary material for: Palladium-catalyzed ring-opening reactions of cyclopropanated 7-oxabenzonorbornadiene with alcohols
Source: Beilstein J Org Chem. 2016 Oct 14;12:2189–96. doi: 10.3762/bjoc.12.209 (PMC5082584; doi:10.3762/bjoc.12.209)
Supplement: File 1 — Experimental. [file Beilstein_J_Org_Chem-12-2189-s001.pdf]

Supporting Information

for

Palladium-catalyzed ring-opening reactions of cyclopropanated 7-oxabenzonorbornadiene with alcohols

Katrina Tait, Oday Alrifai, Rebecca Boutin, Jamie Haner and William Tam*

Address: Guelph-Waterloo Centre for Graduate Work in Chemistry and Biochemistry, Department of Chemistry, University of Guelph, Guelph, Ontario, N1G 2W1, Canada

Email: William Tam - wtam@uoguelph.ca

*Corresponding author

Experimental

Table of Contents

General considerations S2

Experimental procedures and full characterization for key compounds **8a–j** S2

General procedure for the palladium-catalyzed ring-opening reaction of cyclopropanated 7-oxabenzonorbonradiene with alcohols S3

Characterization data for compounds **11a–q** S3–S8

General considerations

All ring-opening reactions were carried out under inert atmospheric conditions. All glassware was oven dried overnight before use. Commercial reagents were all used as received from their respective suppliers. Flash column chromatography was performed on 230–400 mesh silica gel purchased from Silicycle. Analytical TLC was performed on pre-coated silica gel 250 μm 60 F254 aluminum plates purchased from Silicycle. TLC visualization was carried out under UV light and *p*-anisaldehyde stain. Infrared samples were acquired as solids or as neat oils on a Bruker ALPHA platinum single reflection diamond ATR spectrophotometer and are reported in wave numbers (cm^{-1}). ^{1}H and ^{13}C NMR spectra were recorded on a Bruker Avance 400 MHz spectrometer ($CDCl_3$: δ 7.24 ppm (^{1}H at 400 MHz) or δ 77.0 ppm (^{13}C at 100 MHz)). HRMS analyses were performed at the Queen's Mass Spectrometry and Proteomics Unit, Kingston, Ontario. The samples were ionized by electron impact (EI) and detection of the ions was performed by time of flight (TOF).

Experimental procedures and full characterization data for previously reported key compounds

For complete experimental procedures and full characterization data of all starting materials **8a–j** see our previous report on the synthesis of cyclopropanated 7-oxabenzonorbornadienes.[1]

General procedure for the palladium-catalyzed ring-opening reaction of cyclopropanated 7-oxabenzonorbonradiene with alcohols

In a small screw-cap vial containing a stir-bar under an inert atmosphere, catalyst was added (0.1 equiv). Cyclopropanated oxabenzonorbornadiene (30 mg, 1.0 equiv, 0.1-0.3 mmol) was dissolved in alcohol (0.4 mL) or in a mixture of alcohol (0.4 mL) and toluene (0.4 mL) and added to the reaction. The vial was sealed and secured tightly with polytetrafluoroethylene (PTFE) thread-seal tape and paraffin film. The reaction was heated to either 60 °C or 90 °C with continuous stirring for 1–14 days. The crude product was directly loaded onto a chromatography column and purified (EtOAC/hexanes).

2-(Methoxymethyl)naphthalene, 11a (Table 2, entry 11): Yield: 89% (28.2 mg, 0.16 mmol); clear oil; R_f = 0.33 (EtOAc/hexanes, 1:9); ^{1}H NMR ($CDCl_3$, 400 MHz) δ: 7.89-7.86 (m, 3H), 7.83 (br s, 1H), 7.53-7.49 (m, 3H), 4.67 (s, 2H), 3.47 (s, 3H); ^{13}C NMR ($CDCl_3$, 100 MHz) δ: 135.7, 133.3,133.0, 128.2, 127.9, 127.7, 126.4, 126.1, 125.9, 125.7, 74.8, 58.2. Spectral data are consistent with those previously reported. [2]

2-(Methoxymethyl)-1,4-dimethylnaphthalene, 11b (Table 3, entry 1): Yield: 40% (14.6 mg, 0.073 mmol); yellow solid; R_f = 0.39 (EtOAc/hexanes, 1:9); ^{1}H NMR ($CDCl_3$, 400 MHz) δ: 8.10-8.08 (m, 1H), 8.00-7.97 (m, 1H), 7.54-7.49 (m, 2H), 7.31 (s, 1H), 4.63 (s, 2H), 3.43 (s, 3H), 2.66 (s, 3H), 2.64 (s, 3H). ^{13}C NMR ($CDCl_3$, 100 MHz) δ: 133.0, 132.5, 132.4, 132.0, 130.7, 128.0, 125.5, 125.3, 124.7, 124.6, 73.4, 58.2, 19.3, 14.0. Spectral data are consistent with those previously reported.[3]

1,4-Dimethoxy-6-(methoxymethyl)naphthalene, 11c (Table 3, entry 2): Yield: 5% (1.8 mg, 0.0078 mmol); off-white solid; R_f = 0.29 (EtOAc/hexanes, 1:9); ^{1}H NMR ($CDCl_3$, 400 MHz) δ: 8.17 (d, J = 8.6 Hz, 1H), 8.13-8.12 (m, 1H), 7.48 (dd, J = 8.6, 1.7 Hz, 1H), 6.68 (ABq, J_{AB} = 8.4 Hz, $\Delta\delta_{AB}$ = 5.7 Hz, 2H), 4.62 (s, 2H), 3.941 (s, 3H), 3.938 (s, 3H), 3.39 (s, 3H); ^{13}C NMR ($CDCl_3$, 100 MHz) δ: 149.5, 135.7, 126.1,125.9, 125.7, 122.2, 120.7, 103.5, 103.2, 75.0, 58.0, 55.7. Spectral data are consistent with those previously reported.[3,4]

2,3-Dimethoxy-6-(methoxymethyl)naphthalene, 11d (Table 3, entry 3): Yield: 46% (14.6 mg, 0.063 mmol); off-white solid; mp 99-101°C; R_f = 0.34 (EtOAc/hexanes, 1:9); IR (ν, cm^{-1}): 3054, 3006, 2975, 2923, 2810, 1489, 1158, 1104, 856. ^{1}H NMR (400 MHz, $CDCl_3$) δ: 7.66 (d, J = 8.3 Hz, 1H), 7.62 (s, 1H), 7.30 (dd, J = 8.3 Hz, 1.7 Hz, 1H), 7.10 (d, J = 1.2 Hz, 2H), 4.56 (s, 2H), 3.98 (d, J = 1.2 Hz, 6H), 3.40 (s, 3H); ^{13}C NMR ($CDCl_3$, 100 MHz) δ: 149.61, 149.47, 133.97, 129.00, 128.69, 126.58, 125.29, 124.24, 106.31, 106.16, 74.94, 58.06, 55.87 (2C); HRMS (EI) calcd. for $C_{14}H_{16}O_3$ (M^+): 232.1099; found: 232.1089.

2,3-Dibromo-6-(methoxymethyl)naphthalene, 11e (Table 3, entry 4): Yield: 37% (12.0 mg, 0.036 mmol); beige solid; R_f = 0.34 (EtOAc/hexanes, 1:9); ^{1}H NMR ($CDCl_3$, 400 MHz) δ: 8.09 (s, 1H), 8.08 (s, 1H), 7.69 (d, J = 8.5 Hz, 1H), 7.64 (s, 1H), 7.46 (dd, J = 8.5 Hz, 1.6 Hz, 1H), 4.57 (s, 2H), 3.42 (s, 3H); ^{13}C NMR ($CDCl_3$, 100 MHz) δ: 137.4, 133.0, 132.6, 132.2, 132.1, 127.1, 126.9, 125.0, 122.2, 74.4, 58.4. Spectral data are consistent with those previously reported. [3]

3-(Methoxymethyl)-1-methylnaphthalene, 11f (Table 4, entry 2): Yield: 41% (13.7 mg, 0.074 mmol); yellow oil; R_f = 0.43 (EtOAc/hexanes, 1:9); ^{1}H NMR ($CDCl_3$, 400 MHz) δ: 7.98-

7.96 (m, 1H), 7.84-7.81 (m, 1H), 7.64 (s, 1H), 7.53-7.45 (m, 2H), 7.31 (s, 1H), 4.58 (s, 2H), 3.42 (s, 3H), 2.69 (s, 3H); ^{13}C NMR ($CDCl_3$, 100 MHz) δ: 135.2, 134.7, 133.5, 132.2, 128.5, 126.4, 125.8, 125.7, 125.0, 124.0, 74.9, 58.2, 19.4. Spectral data are consistent with those previously reported. [3]

3-(Methoxymethyl)-1-ethylnaphthalene, 11g (Table 4, entry 4): Yield: 65% (20.7 mg, 0.10 mmol); clear oil; R_f = 0.43 (EtOAc/hexanes, 1:9); IR (ν, cm^{-1}): 3066, 3053, 2963, 2850, 1097, 872, 744. ^{1}H NMR (400 MHz, $CDCl_3$) δ: 8.04-8.01 (m, 1H), 7.84-7.81 (m, 1H), 7.63 (s, 1H), 7.51-7.43 (m, 2H), 7.32 (s, 1H), 4.59 (s, 2H), 3.42 (s, 3H), 3.10 (q, *J* = 7.5 Hz, 2H), 1.38 (t, *J* = 7.5 Hz, 3H); ^{13}C NMR ($CDCl_3$, 100 MHz) δ: 140.70, 135.31, 133.73, 131.38, 128.75, 125.71, 125.67, 125.00, 124.71, 123.68, 74.94, 58.20, 25.93, 15.02; HRMS (EI) calcd. for $C_{14}H_{16}O$ (M^+): 200.1201; found: 200.1209.

3-(Methoxymethyl)-1-*tert*-butylnaphthalene, 11h (Table 4, entry 5): Yield: 47% (14.2 mg, 0.062 mmol); yellow solid; mp.. 38-39°C R_f = 0.47 (EtOAc/hexanes, 1:9); IR (ν, cm^{-1}): 3053, 2957, 1461, 1396, 1366, 1100, 876, 751; ^{1}H NMR ($CDCl_3$, 400 MHz) δ: 8.41 (br d, *J* = 8.4 Hz, 1H), 7.85-7.82 (m, 1H), 7.63 (br s, 1H), 7.47-7.40 (m, 3H), 4.58 (s, 2H), 3.43 (s, 3H), 1.61 (s, 9H); ^{13}C NMR ($CDCl_3$, 100 MHz) δ: 146.5, 135.0, 134.6, 131.1, 129.6, 126.9, 126.0, 124.9, 124.6, 123.2, 75.1, 58.2, 36.1, 31.8; HRMS (EI) calcd for $C_{16}H_{20}O$ (M^+): 228.1514; found 228.1519.

3-(Methoxymethyl)-1-acetylnaphthalene, 11i (Table 4, entry 6): Yield: 29% (9.3 mg, 0.043 mmol); yellow oil; R_f = 0.17 (EtOAc/hexanes, 1:9); IR (ν, cm^{-1}): 3053, 2923, 2850, 2822, 1676, 1239, 1098, 753. ^{1}H NMR (400 MHz, $CDCl_3$) δ: 8.68 (d, *J* = 8.3 Hz, 1H), 7.91 (s, 2H), 7.84 (dd, *J* = 8.2 Hz, 1.1 Hz, 1H), 7.59-7.50 (m, 2H), 4.63 (s, 2H), 3.44 (s, 3H), 2.75 (s, 3H); ^{13}C NMR

($CDCl_3$, 100 MHz) δ: 201.90, 135.88, 134.28, 133.93, 131.30, 129.68, 128.40 (2C), 128.03, 126.72, 125.95, 74.25, 58.37, 30.07; HRMS (EI) calcd. for $C_{14}H_{14}O_2$ (M^+): 214.0994; found: 214.0985.

3-(Methoxymethyl)-1-(methoxycarbonyl)naphthalene, 11j (Table 4, entry 7): Yield: 23% (8.1 mg, 0.035 mmol); clear oil; R_f = 0.43 (EtOAc/hexanes, 1:9); IR (ν, cm^{-1}): 3055, 2988, 2820, 1714, 1242, 1196, 1100, 795. ^1H NMR (400 MHz, $CDCl_3$) δ: 8.87 (d, *J* = 8.6 Hz, 1H), 8.16, (d, *J* = 1.6 Hz, 1H), 7.95 (s, 1H), 7.85 (d, *J* = 8.2 Hz, 1H), 7.61-7.50 (m, 2H), 4.62 (s, 2H), 3.99 (s, 3H), 3.43 (s, 3H); ^{13}C NMR ($CDCl_3$, 100 MHz) δ: 167.90, 134.51, 133.85, 131.58, 130.85, 130.03, 128.54, 127.72, 127.37, 126.48, 125.76; HRMS (EI) calcd. for $C_{14}H_{14}O_3$ (M^+): 230.0943; found: 230.0939.

2-(Ethoxymethyl)naphthalene, 11k (Table 5, entry 2): Yield: 85% (31.5mg, 0.17 mmol); clear oil; R_f = 0.40 (EtOAc/hexanes, 1:9); ^1H NMR (400 MHz, $CDCl_3$) δ: 7.83-7.81 (m, 3H), 7.78 (br s, 1H), 7.48-7.44 (m, 3H), 4.67 (s, 2H), 3.58 (q, *J* = 7.0 Hz, 2H), 1.27 (t, *J* = 7.0 Hz, 3H); ^{13}C NMR (100 MHz, $CDCl_3$) δ: 136.1, 133.3, 133.0, 128.1, 127.9, 127.7, 126.3, 126.0, 125.8, 125.7, 72.8, 65.8, 15.3. Spectral data are consistent with those previously reported. [2]

2-(Butoxymethyl)naphthalene, 11l (Table 5, entry 3): Yield: 68% (29.3mg, 0.14 mmol); clear oil; R_f = 0.40 (EtOAc/hexanes, 1:19); ^1H NMR (400 MHz, $CDCl_3$) δ: 7.83-7.81 (m, 3H), 7.77 (br s, 1H), 7.48-7.44 (m, 3H), 4.66 (s, 2H), 3.51 (t, *J* = 6.6 Hz, 2H), 1.66-1.58 (m, 2H), 1.45-1.38 (m, 2H), 0.92 (t, *J* = 7.4 Hz, 3H); ^{13}C NMR (100 MHz, $CDCl_3$) δ: 136.3, 133.3, 133.0, 128.1,

127.9, 127.7, 126.3, 126.0, 125.8, 125.7, 73.0, 70.3, 31.9, 19.4, 14.0. Spectral data are consistent with those previously found. [3,5]

2-[(2-methoxyethoxy)methyl]naphthalene, 11m (Table 5, entry 4): Yield: 80% (33.9mg, 0.16 mmol); yellow oil; R_f = 0.19 (EtOAc/hexanes, 1:9); IR (ν, cm^{-1}): 3054, 2875, 1509, 1363, 1344, 1199, 1170, 1124, 1100, 1047, 855, 817, 752. ^{1}H NMR (400 MHz, $CDCl_3$) δ: 7.83-7.80 (m, 3H), 7.78 (br s, 1H), 7.49-7.44 (m, 3H), 4.73 (s, 2H), 3.66-3.63 (m, 2H), 3.60-3.57 (m, 2H), 3.40 (s, 3H); ^{13}C NMR (100 MHz, $CDCl_3$) δ: 135.7, 133.3, 133.0, 128.2, 127.9, 127.7, 126.6, 126.1, 125.9, 125.8, 73.4, 72.0, 69.3, 59.1; HRMS (EI) calcd for $C_{14}H_{16}O_2$ (M^+): 216.1150; found: 216.1158.

2-[(2-methylpropoxy)methyl]naphthalene, 11n (Table 5, entry 5): Yield: 60% (27.6mg, 0.13 mmol); clear oil; R_f = 0.51(EtOAc/hexanes, 1:9); IR (ν, cm^{-1}): 3055, 2956, 2927, 2871, 1509, 1469, 1366, 1124, 1097, 854, 817, 750; ^{1}H NMR (400 MHz, $CDCl_3$) δ: 7.88-7.85 (m, 3H), 7.82 (s, 1H), 7.53-7.49 (m, 3H), 4.71 (s, 2H), 3.31 (d, *J* = 6.7 Hz, 2H), 1.98 (app. non, *J* = 6.7 Hz, 1H), 0.98 (d, *J* = 6.7 Hz, 6H); ^{13}C NMR (100 MHz, $CDCl_3$) δ: 136.3, 133.3, 132.9, 128.1, 127.9, 127.7, 126.2, 126.0, 125.8 (2C), 77.4, 73.1, 28.6, 19.5 (2C); HRMS (EI) calcd for $C_{15}H_{18}O$ (M^+): 214.1358; found: 214.1352.

2-(Isoproproxymethyl)naphthalene, 11o (Table 5, entry 6): Yield: 41% (17.1mg, 0.085 mmol); clear oil; R_f = 0.41 (EtOAc/hexanes, 1:9); ^{1}H NMR (400 MHz, $CDCl_3$) δ: 7.82-7.80 (m, 3H), 7.78 (s, 1H), 7.48-7.43 (m, 3H), 4.67 (s, 2H), 3.73 (sept, *J* = 6.1 Hz, 1H), 1.24 (d, *J* = 6.1

Hz, 6H); ^{13}C NMR (100 MHz, $CDCl_3$) δ: 136.6, 133.4, 132.9, 128.1, 127.9, 127.7, 126.1, 126.0, 125.8, 125.7, 71.0, 70.2, 22.2; Spectral data are consistent with those previously reported. [3]

2-(*tert*-Butoxymethyl)naphthalene, 11p (Table 5, entry 7): Yield: 56% (23.9mg, 0.11 mmol); clear oil; R_f = 0.40 (EtOAc/hexanes 1:9); ^{1}H NMR (400 MHz, $CDCl_3$) δ: 7.83-7.79 (m, 4H), 7.47-7.41 (m, 3H), 4.61 (s, 2H), 1.34 (s, 9H); ^{13}C NMR (100 MHz, $CDCl_3$) δ: 137.3, 133.4, 132.7, 127.9, 127.8, 127.6, 125.82, 125.78, 125.73, 125.5, 73.6, 64.2, 27.7. Spectral data are consistent with those previously reported.[2]

2-(Cyclohexoxymethyl)naphthalene, 11q (Table 5, entry 8): Yield: 63% (34.7mg, 0.14 mmol); R_f = 0.44 (EtOAc/hexanes 1:9); ^{1}H NMR (400 MHz, $CDCl_3$) δ: 7.82-7.79 (m, 3H), 7.77 (br s, 1H), 7.48-7.43 (m, 3H), 4.70 (s, 2H), 3.41-3.36 (m, 1H), 1.99-1.96 (m, 2H), 1.77-1.72 (m, 2H), 1.55-1.51 (m, 1H), 1.42-1.33 (m, 2H), 1.29-1.21 (m, 3H).^{13}C NMR (100 MHz, $CDCl_3$) δ: 136.8, 133.3, 132.9, 128.1, 127.9, 127.7, 126.1, 126.0, 125.8, 125.7, 77.0, 69.8, 32.3, 25.9, 24.2. Spectral data are consistent with those previously reported.[3,6]

References

1. McKee, M.; Haner, J.; Carlson, E.; Tam, W. *Synthesis* **2014**, *46*, 1518-1524. doi:10.1055/s-0033-1340918
2. Guan, B.-T.; Xiang, S.-K.; Wang, B.-Q.; Sun, Z.-P.; Wang, Y.; Zhao, K.-Q.; Shi, Z.-J. *J. Am. Chem. Soc.* **2008**, *130*, 3268-3269. doi:10.1021/ja710944j
3. Tigchelaar, A.; Haner, J.; Carlson, E.; Tam, W. *Synlett* **2014**, *25*, 2355-2359. doi:10.1055/2-0034-1378581

4. Halton, B.; Kay, A.J.; Zhi-mei, Z.; Boese, R.; Haumann, T. *J. Chem. Soc., Perkin Trans. 1* **1996**, 1445-1452. doi:10.1039/P19960001445
5. Nakata, D.; Sakuma, T.; Kono, K.; Sato. S.; Tani, S.; Kunishima, M. *Chem. Pharm. Bull.* **2001**, *49*, 97-100. doi:10.1248/cpb.49.97
6. Ahern, C.; Darcy, R. *Synth. Commun.* **1998**, *28*, 971-976. doi:10.1080/00397919808003066
