# Supplementary material for: Palladium-catalyzed ring-opening reactions of cyclopropanated 7-oxabenzonorbornadiene with alcohols
Source: Beilstein J Org Chem. 2016 Oct 14;12:2189–96. doi: 10.3762/bjoc.12.209 (PMC5082584; doi:10.3762/bjoc.12.209)
Supplement: File 2 — NMR Spectra. [file Beilstein_J_Org_Chem-12-2189-s002.pdf]

Supporting Information

for

Palladium-catalyzed ring-opening reactions of cyclopropanated 7-oxabenzonorbornadiene with alcohols

Katrina Tait, Oday Alrifai, Rebecca Boutin, Jamie Haner and William Tam*

Address: Guelph-Waterloo Centre for Graduate Work in Chemistry and Biochemistry, Department of Chemistry, University of Guelph, Guelph, Ontario, N1G 2W1, Canada

Email: William Tam - wtam@uoguelph.ca

*Corresponding author

NMR spectra

Table of Contents

^{1}H and ^{13}C NMR spectra for new compounds **11d , g–j, m, n** S2–S8

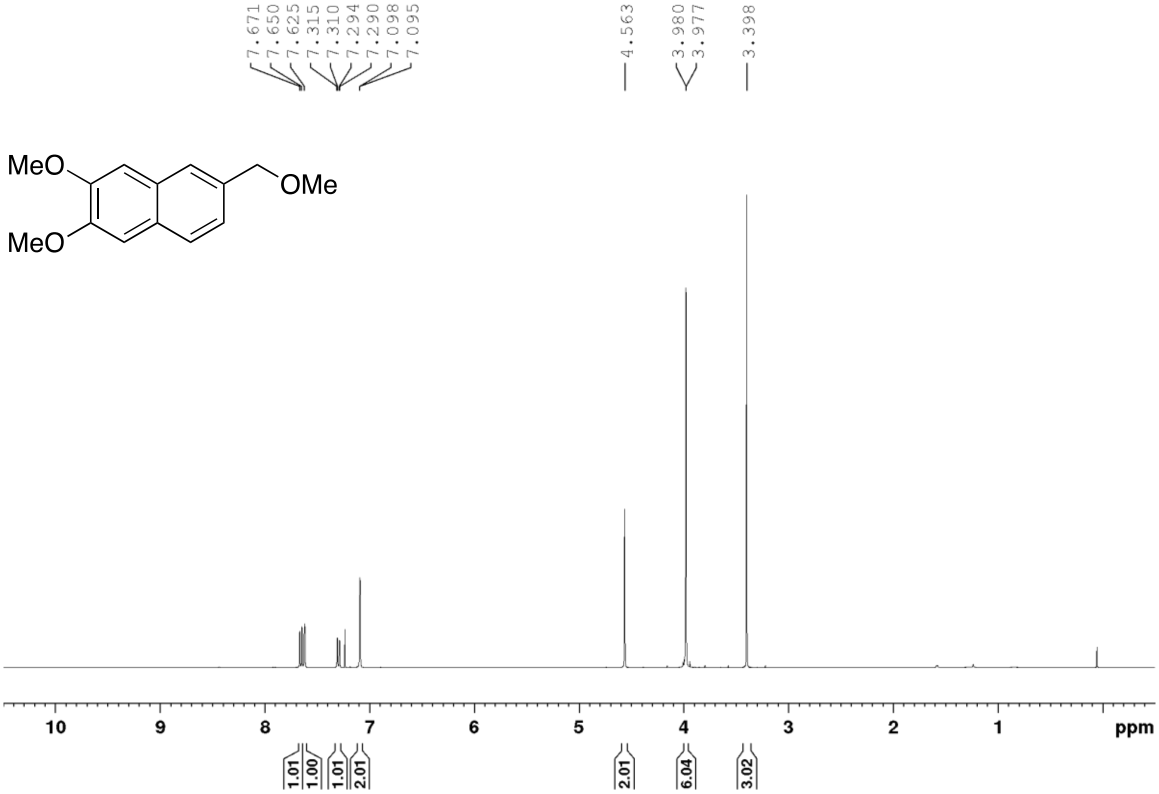

400 MHz ^{1}H NMR spectrum of **11d** in $CDCl_3$

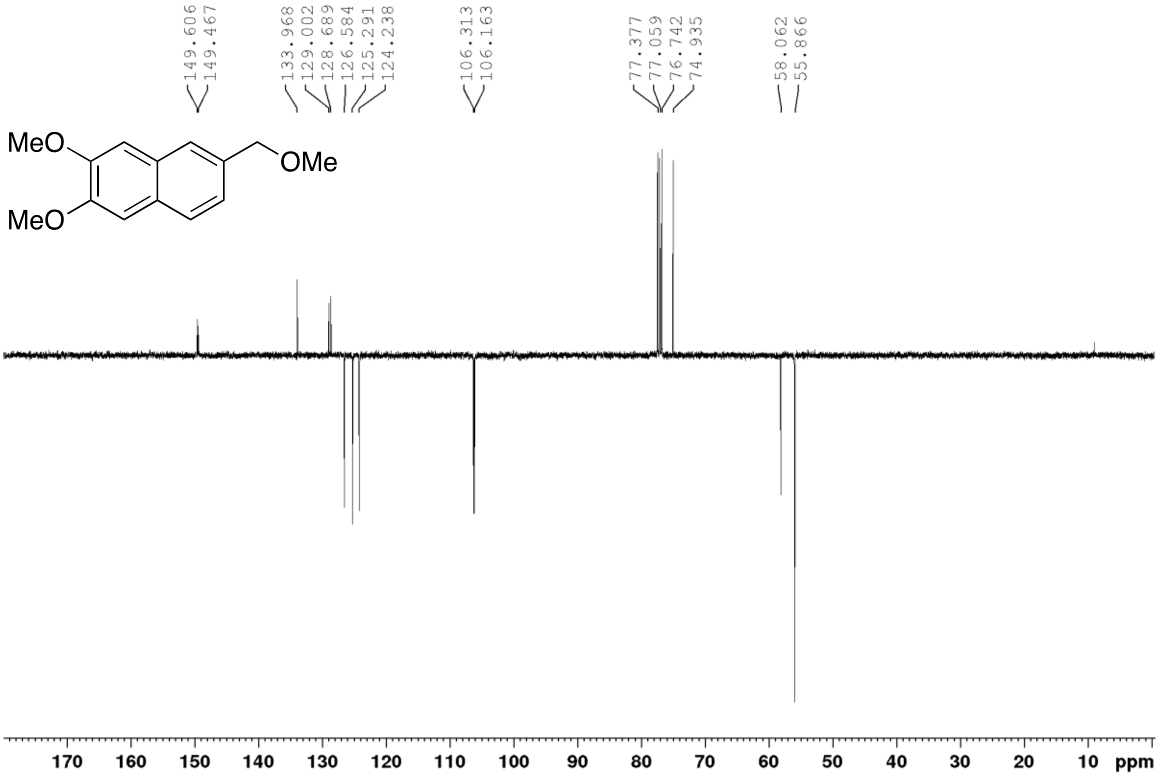

100 MHz ^{13}C NMR spectrum of **11d** in $CDCl_3$

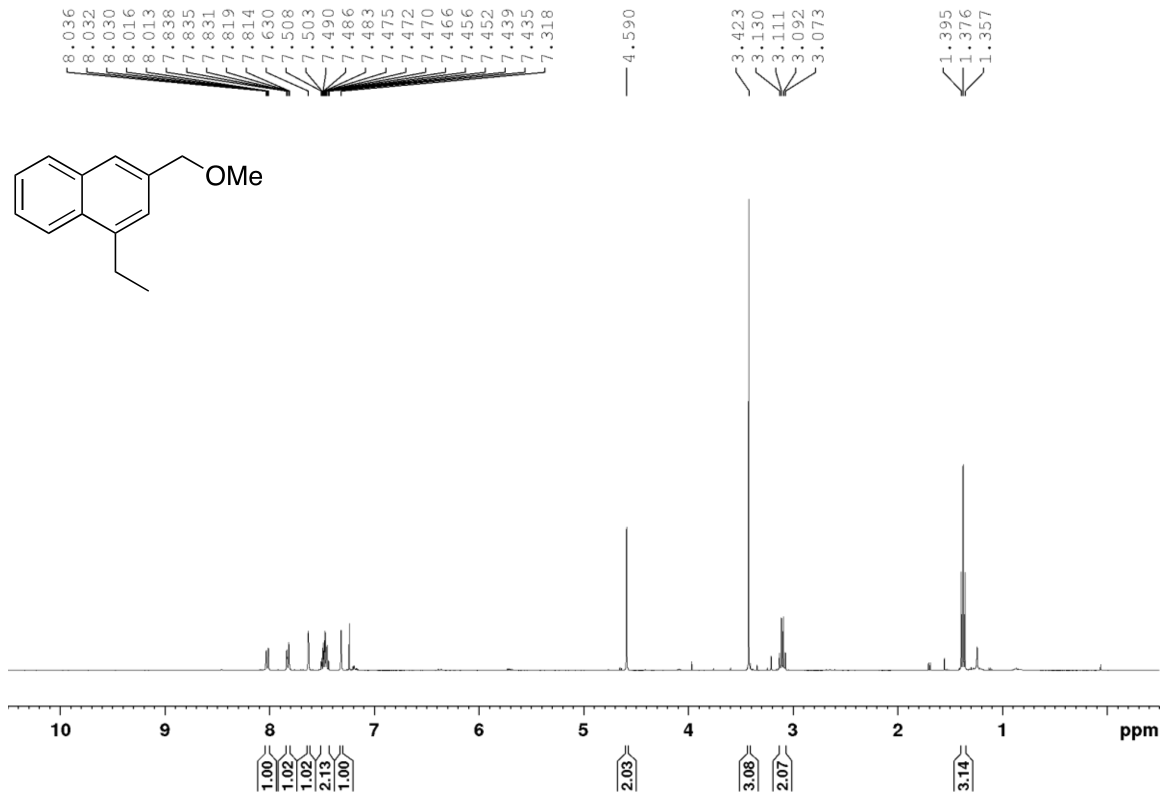

400 MHz ^{1}H NMR spectrum of **11g** with $CDCl_3$

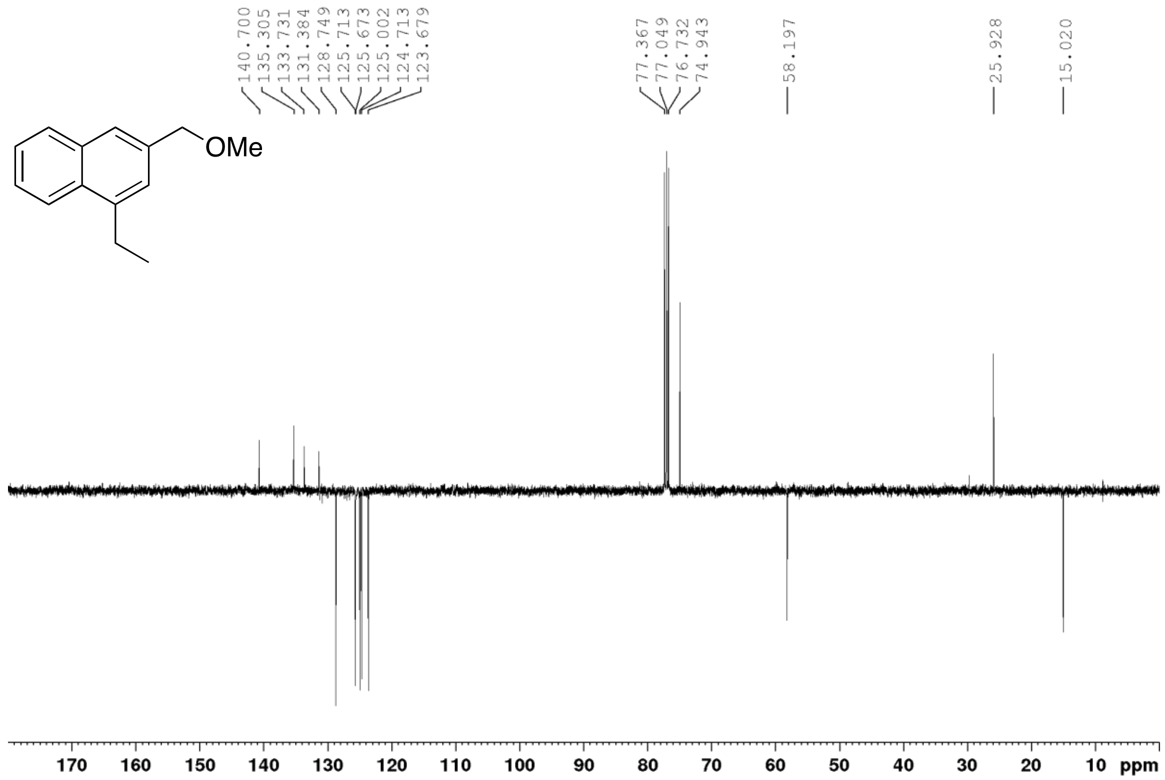

100 MHz ^{13}C NMR spectrum of **11g** with $CDCl_3$

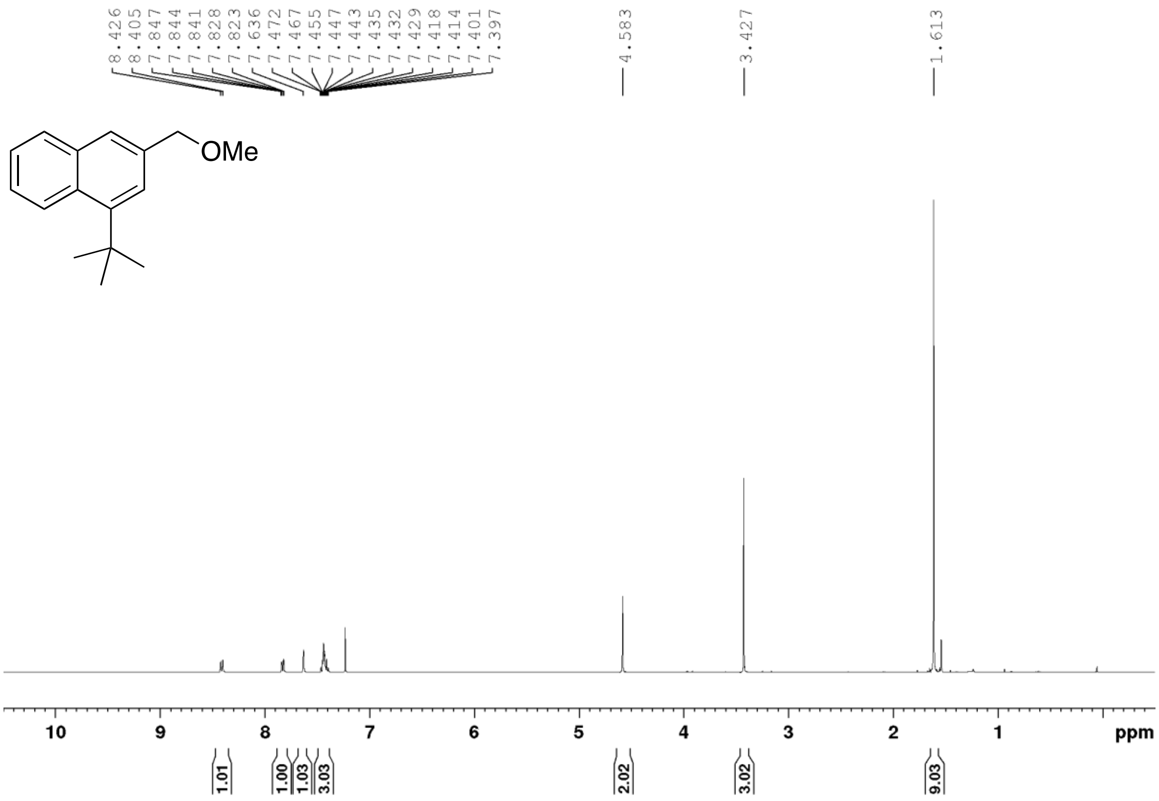

400 MHz ^{1}H NMR spectrum of **11h** in $CDCl_3$

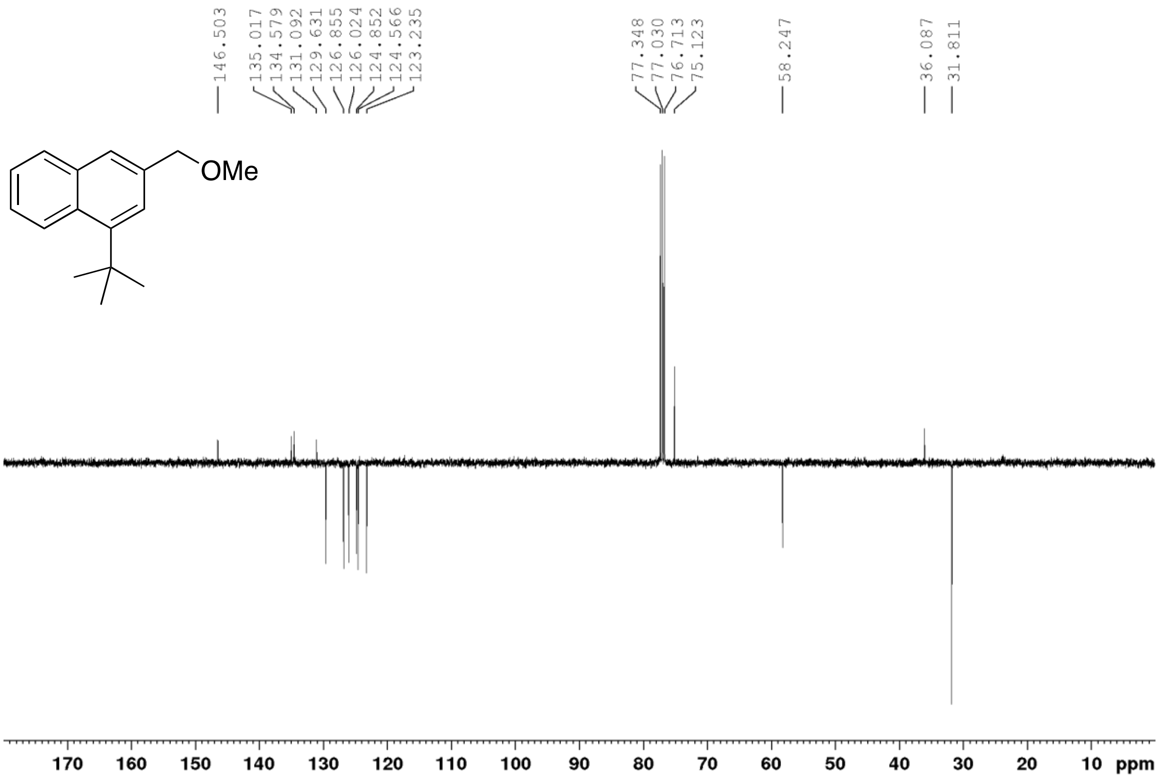

100 MHz ^{13}C NMR spectrum of **11h** in $CDCl_3$

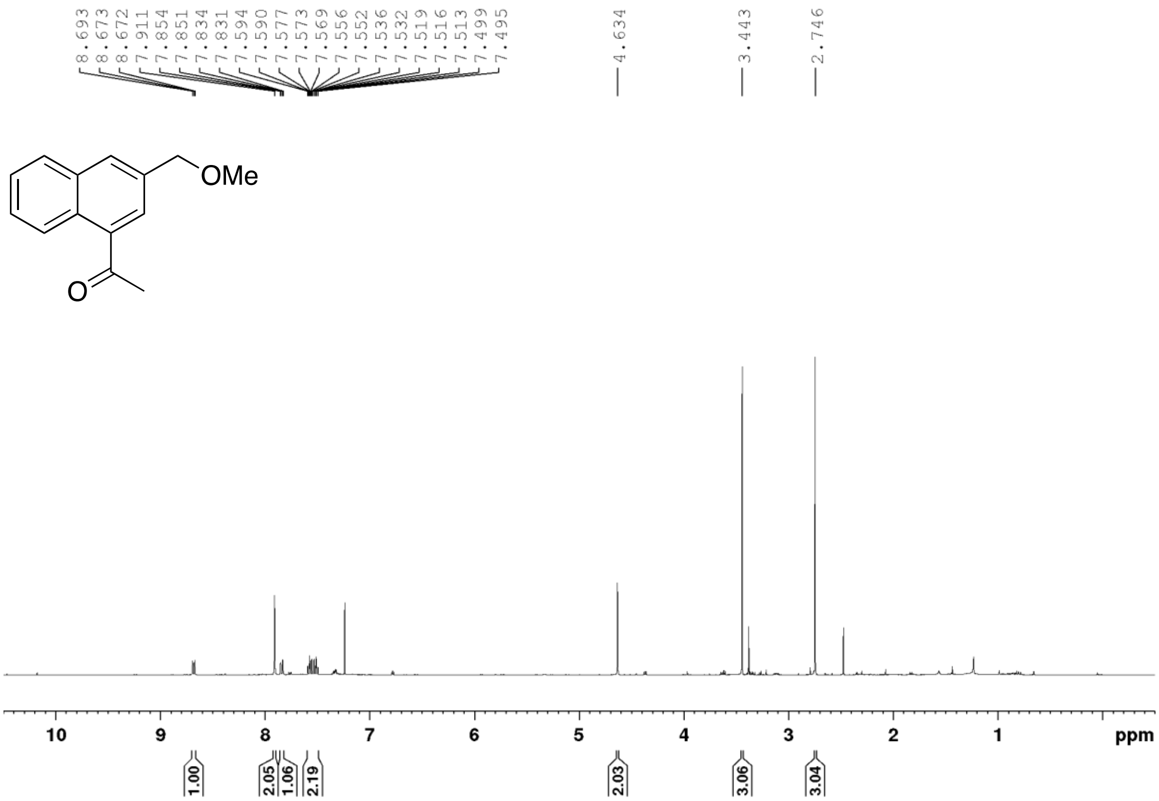

400 MHz ^{1}H NMR spectrum of **11i** in $CDCl_3$

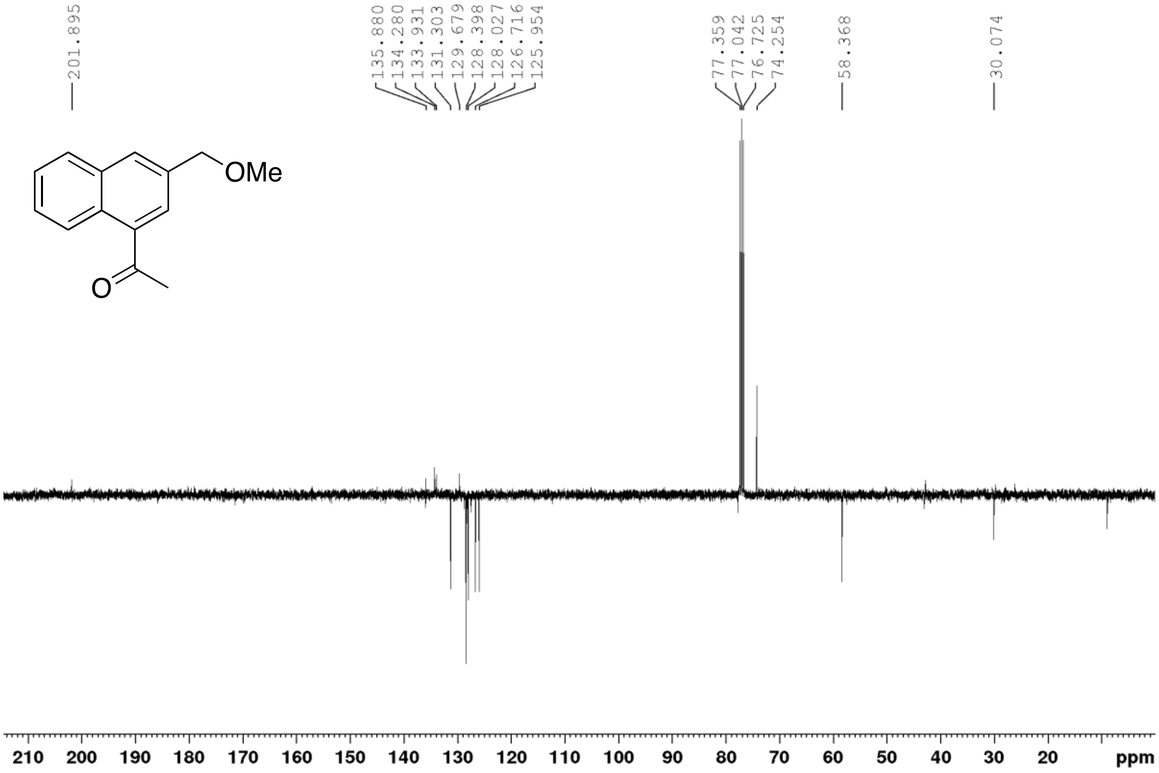

100 MHz ^{13}C NMR spectrum of **11i** in $CDCl_3$

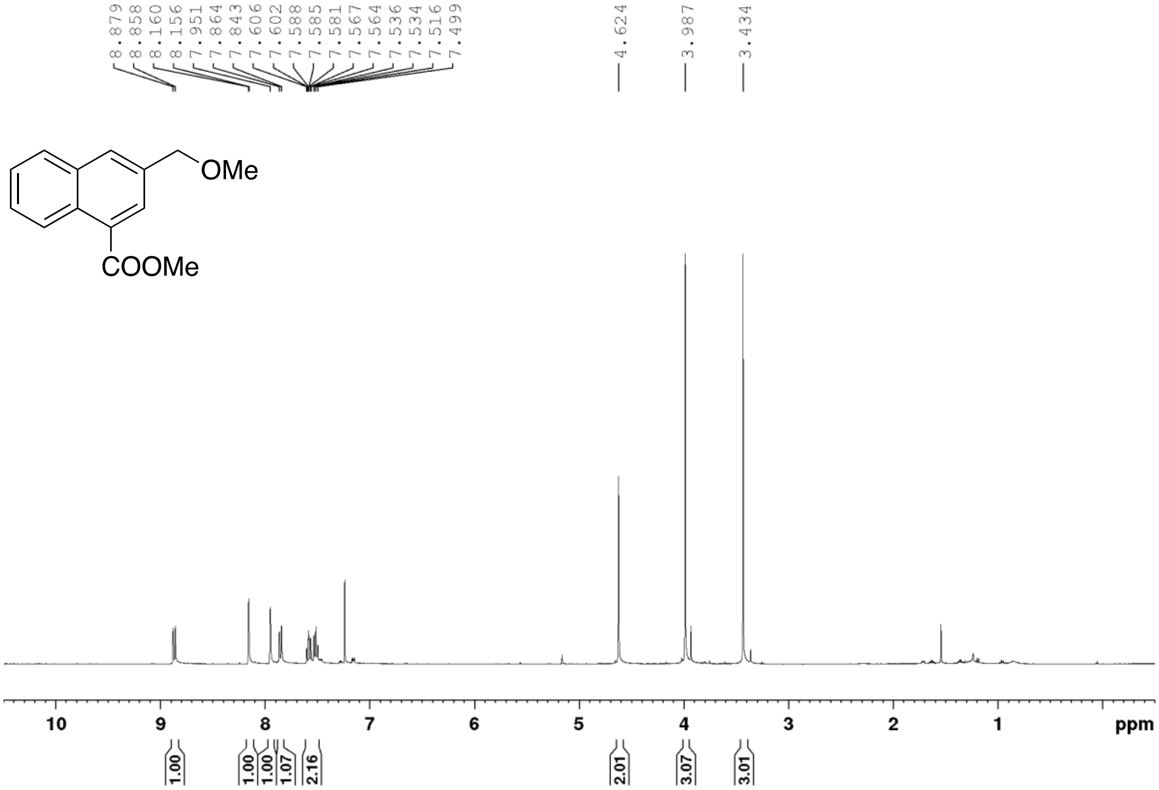

400 MHz ^{1}H NMR spectrum of **11j** in $CDCl_3$

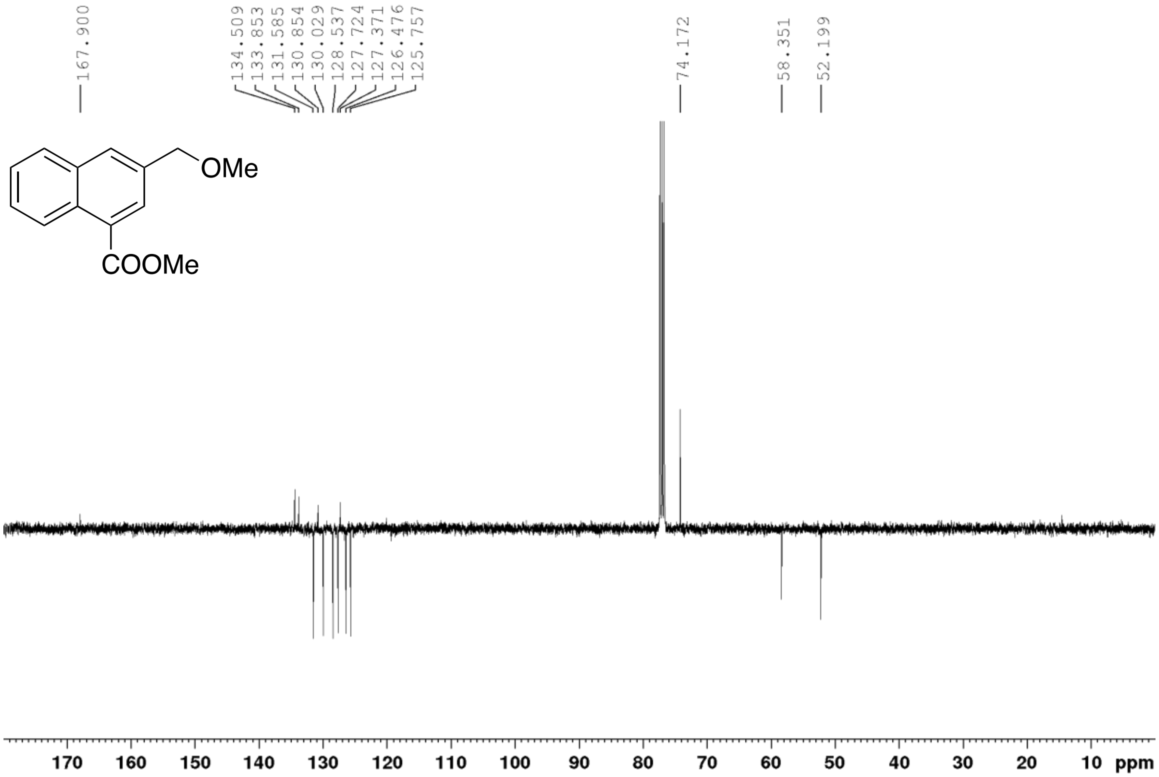

100 MHz ^{13}C NMR spectrum of **11j** in $CDCl_3$

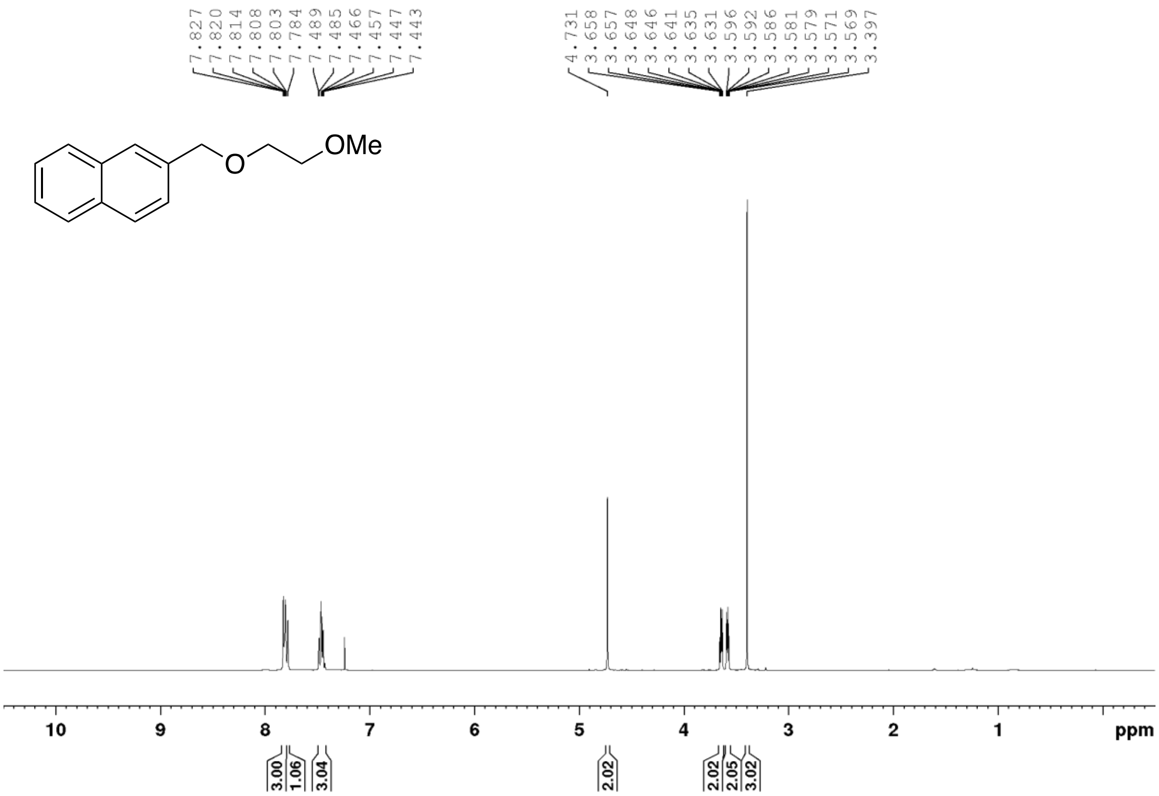

400 MHz ^{1}H spectrum of **11m** in $CDCl_3$

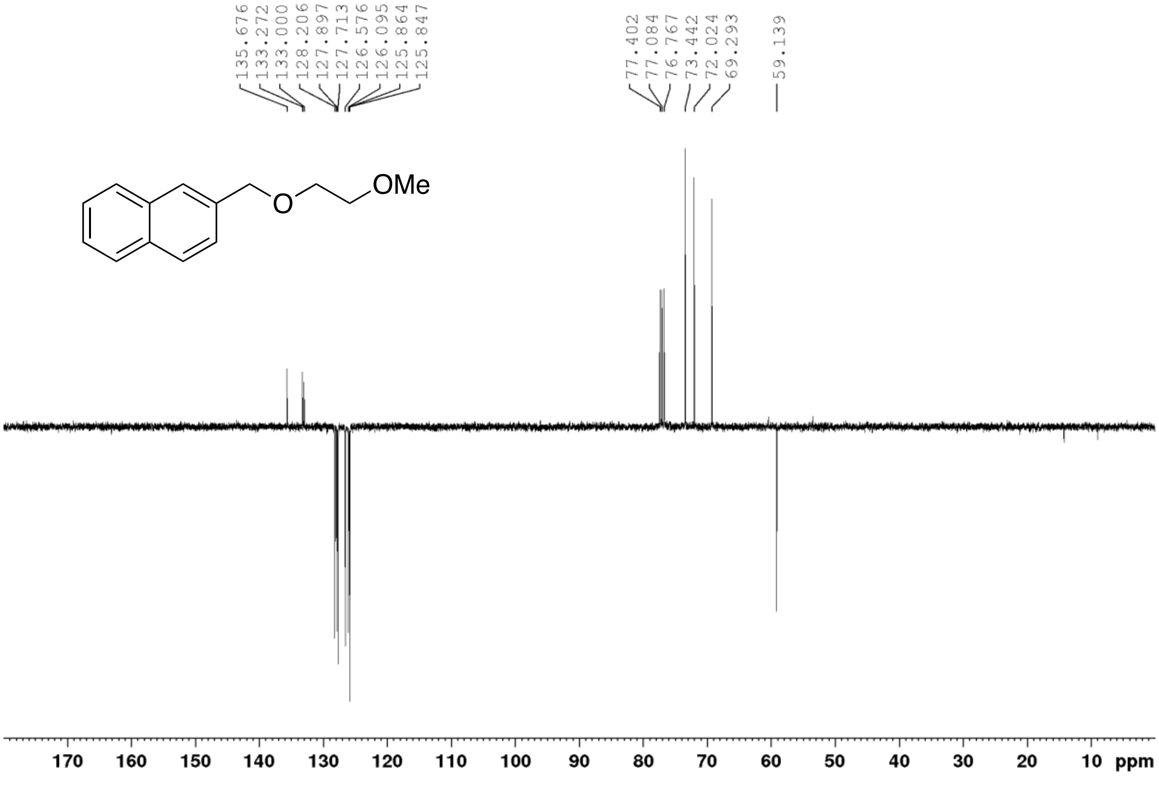

100 MHz ^{13}C NMR spectrum of **11m** in $CDCl_3$

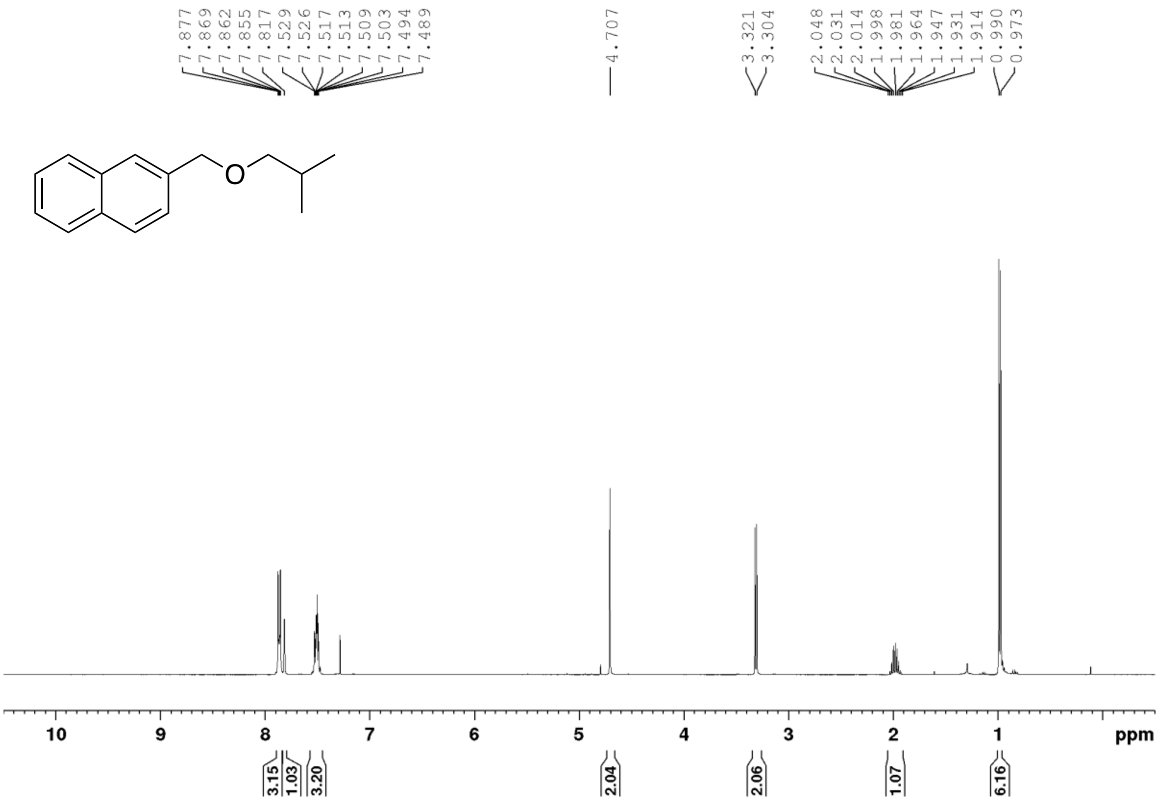

400 MHz ^{1}H NMR spectrum of **11n** in $CDCl_3$

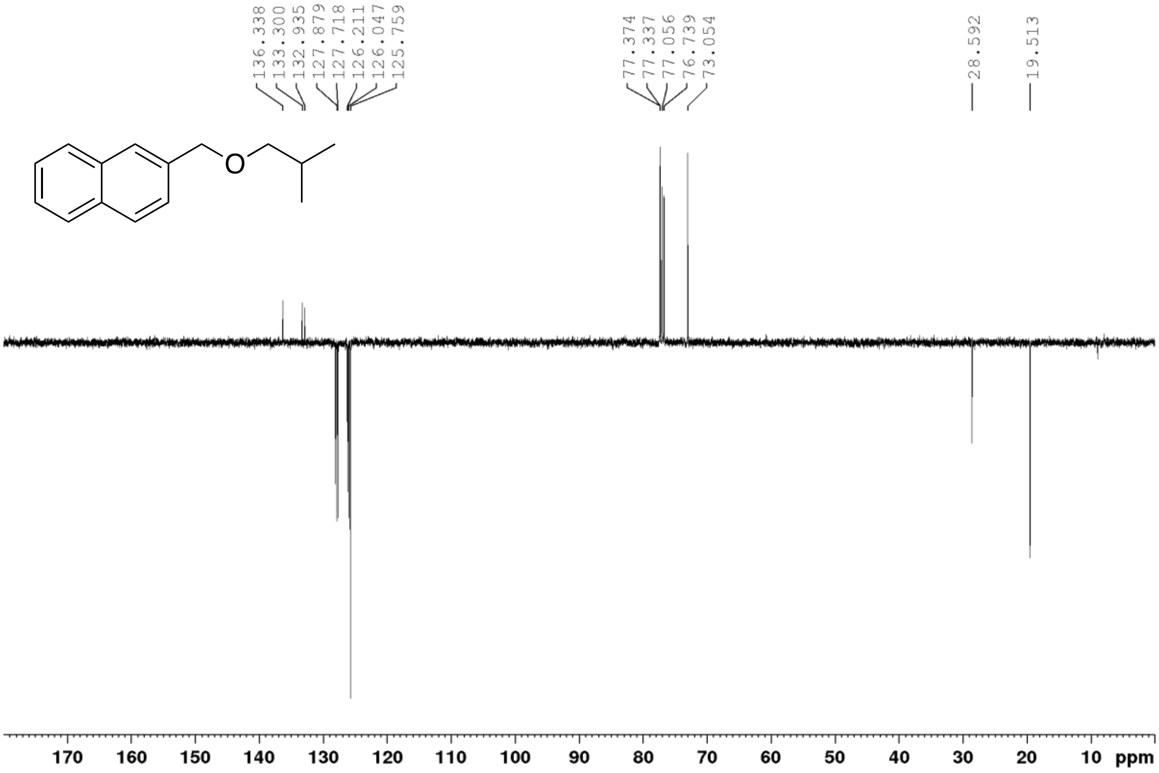

100 MHz ^{13}C NMR spectrum of **11n** in $CDCl_3$
